# Reprogramming Suppresses Premature Senescence Phenotypes of Werner Syndrome Cells and Maintains Chromosomal Stability over Long-Term Culture

**DOI:** 10.1371/journal.pone.0112900

**Published:** 2014-11-12

**Authors:** Akira Shimamoto, Harunobu Kagawa, Kazumasa Zensho, Yukihiro Sera, Yasuhiro Kazuki, Mitsuhiko Osaki, Mitsuo Oshimura, Yasuhito Ishigaki, Kanya Hamasaki, Yoshiaki Kodama, Shinsuke Yuasa, Keiichi Fukuda, Kyotaro Hirashima, Hiroyuki Seimiya, Hirofumi Koyama, Takahiko Shimizu, Minoru Takemoto, Koutaro Yokote, Makoto Goto, Hidetoshi Tahara

**Affiliations:** 1 Department of Cellular and Molecular Biology, Graduate School of Biomedical & Health Sciences, Hiroshima University, Hiroshima, Japan; 2 Department of Biomedical Science, Institute of Regenerative Medicine and Biofunction, Graduate School of Medical Science, Tottori University, Yonago, Japan; 3 Division of Pathological Biochemistry, Faculty of Medicine, Tottori University, Yonago, Japan; 4 Medical Research Institute, Kanazawa Medical University, Kahoku, Ishikawa, Japan; 5 Department of Genetics, Radiation Effects Research Foundation, Hiroshima, Japan; 6 Department of Cardiology, Keio University School of Medicine, Tokyo, Japan; 7 Division of Molecular Biotherapy, The Cancer Chemotherapy Center, Japanese Foundation For Cancer Research, Tokyo, Japan; 8 Department of Advanced Aging Medicine, Chiba University Graduate School of Medicine, Chiba, Japan; 9 Department of Clinical Cell Biology and Medicine, Chiba University Graduate School of Medicine, Chiba, Japan; 10 Division of Orthopedic Surgery & Rheumatology, Tokyo Women's Medical University Medical Center East, Tokyo, Japan; The University of Hong Kong, Hong Kong

## Abstract

Werner syndrome (WS) is a premature aging disorder characterized by chromosomal instability and cancer predisposition. Mutations in *WRN* are responsible for the disease and cause telomere dysfunction, resulting in accelerated aging. Recent studies have revealed that cells from WS patients can be successfully reprogrammed into induced pluripotent stem cells (iPSCs). In the present study, we describe the effects of long-term culture on WS iPSCs, which acquired and maintained infinite proliferative potential for self-renewal over 2 years. After long-term cultures, WS iPSCs exhibited stable undifferentiated states and differentiation capacity, and premature upregulation of senescence-associated genes in WS cells was completely suppressed in WS iPSCs despite *WRN* deficiency. WS iPSCs also showed recapitulation of the phenotypes during differentiation. Furthermore, karyotype analysis indicated that WS iPSCs were stable, and half of the descendant clones had chromosomal profiles that were similar to those of parental cells. These unexpected properties might be achieved by induced expression of endogenous telomerase gene during reprogramming, which trigger telomerase reactivation leading to suppression of both replicative senescence and telomere dysfunction in WS cells. These findings demonstrated that reprogramming suppressed premature senescence phenotypes in WS cells and WS iPSCs could lead to chromosomal stability over the long term. WS iPSCs will provide opportunities to identify affected lineages in WS and to develop a new strategy for the treatment of WS.

## Introduction

Werner syndrome (WS) is a rare human autosomal recessive disorder characterized by early onset of aging-associated diseases, chromosomal instability, and cancer predisposition [1], [2]. Fibroblasts from WS patients exhibit premature replicative senescence [3], and *WRN*, a gene responsible for the disease, encodes a RecQ-type DNA helicase [4]–[7], that is involved in maintenance of chromosome integrity during DNA replication, repair, and recombination [8], [9]. WRN helicase is known to interact with a variety of proteins associated with DNA metabolism including proteins of replication fork progression, base excision repair, and telomere maintenance [8], [9]. The dysfunction of WRN helicase causes defects in telomeric lagging-strand synthesis and telomere loss during DNA replication [10]. Further, it is also reported that telomere loss caused by a defect in WRN helicase involves chromosome end fusions that are suppressed by telomerase [11]. These observations suggest that premature senescence in WS cells reflects defects in telomeric lagging-strand synthesis followed by accelerated telomere loss during DNA replication.

Somatic cell reprogramming follows the introduction of several pluripotency genes including Oct3/4, Sox2, Klf4, c-myc, Nanog and Lin-28 into differentiated cells such as dermal fibroblasts, blood cells, and other cell types [12]–[17]. During reprogramming, somatic cell-specific genes are suppressed, and embryonic stem cell (ESC)-specific pluripotency genes are induced, leading to the generation of iPSCs with undifferentiated states and pluripotency [18]. In addition, ESC-like infinite proliferative potential is directed by induction of the endogenous telomere reverse-transcriptase catalytic subunit (hTERT) gene and the reactivation of telomerase activity during reprogramming [13], [18].

Recently, Cheung et al. demonstrated that cells from WS patients were successfully reprogrammed into iPSCs with restored telomere function, suggesting that the induction of hTERT during reprogramming suppresses telomere dysfunction in WS cells lacking *WRN* [19]. However, the effects of long-term culture on the undifferentiated states, self-renewal abilities, and differentiation potentials of WS iPSCs remain unknown. In a previous study, progressive telomere shortening and loss of self-renewal ability were observed in iPSCs from dyskeratosis congenital patient cells in a long-term culture [20], warranting the evaluation of the properties of patient cell-derived iPSCs with telomere dysfunctions over the long term.

In this study, we cultured WS iPSCs with self-renewal capacity and infinite proliferative potential for over 2 years and reported similar properties to those of normal iPSCs including undifferentiated states and differentiation ability. Notably, WS iPSCs maintained stable karyotypes and their potential to recapitulate premature senescence phenotypes during differentiation over the long term. The present data demonstrate that reprogramming suppresses premature senescence phenotypes in WS cells by reversing the aging process and restoring telomere maintenance over the long term.

## Materials and Methods

### Cell lines

WS patients were diagnosed on the basis of clinical symptoms and *WRN* gene mutations. A0031 WS patient fibroblasts from a 37-year-old male were obtained from Goto Collection of RIKEN Bioresource Center (https://www.brc.riken.jp/lab/cell/english/index_gmc.shtml) [21], and WSCU01 patient fibroblasts were isolated from a 63-year-old Japanese male who was diagnosed at Chiba University. Both fibroblast isolates had type 4/6 heterozygous mutations. TIG-3 human fetal lung-derived fibroblast cells and WS patient-derived fibroblasts were used to generate iPSC lines. PLAT-A cells (kindly provided from Dr. Toshio Kitamura) were used to produce retroviruses [22]. SNL 76/7 (SNL) cells (DS pharma biomedical) were used as feeder layers for reprogramming of fibroblasts and maintenance of iPSCs. The human fibroblast-derived iPSC line iPS-TIG114-4f1 was obtained from the National Institute of Biomedical Innovation [23].

PLAT-A cells, TIG-3 fibroblasts, TIG-114 fibroblasts from the 36-year-old male, and SNL cells were grown in the Dulbecco's modified Eagle's medium (DMEM; Sigma) supplemented with 10% fetal bovine serum (FBS; Hyclone) and antibiotics (Invitrogen). WS fibroblasts were maintained on collagen-coated dishes (Nitta Gelatin), SNL cells were maintained on gelatin-coated dishes (Nitta Gelatin), and iPSCs were maintained in the ES medium comprising Knockout DMEM (Invitrogen) supplemented with 20% Knockout Serum Replacement (Invitrogen), glutamine, non-essential amino acids, β-mercaptoethanol and 4-ng/ml basic FGF. All cells were maintained at 37°C under 5% CO_2_ atmosphere.

### Generation of iPSCs

The generation of iPSCs was performed as described previously [13]. Briefly, 2×10^6^ PLAT-A cells were plated in T25 flasks (Biocoat, BD Falcon), and were transfected with 4 µg pMXs-OCT3/4, SOX2, KLF-4, and c-myc (Addgene) 1 day later. Twenty-four hours after transfection, the culture medium was replaced with a fresh medium and cells were incubated for 24 h prior to harvest of viral supernatants. Viral supernatants containing Yamanaka factors were combined in even ratios.

For reprogramming experiments, 3×10^5^ fibroblasts were seeded on 60-mm dishes and were infected with viral supernatants containing Yamanaka factors in the presence of 8 µg/ml polybrene 1 day later. Four days after infection, fibroblasts were harvested, and 1×10^5^ cells were reseeded onto mitomycin C-inactivated SNL feeder layers on 100-mm dishes. Twenty-four hours after reseeding, the medium was replaced with the ES medium, and cultures were maintained by replacing the medium every other day. Approximately 30 days after retroviral transduction, emerging iPSC colonies with ESC colony-like flat and round shapes were picked up by mechanical dissection and were plated onto fresh feeder layers on 4-well plates (Thermo Scientific Nunc). Subsequently, iPSC lines were established by successive passages onto fresh feeder layers with split ratios between 1∶3 and 1∶5 using dispase (Roche Applied Science).

### Alkaline phosphatase activity

Undifferentiated states of emerging colonies were examined using alkaline phosphatase staining. After formalin fixation, colonies were stained with reaction buffer containing 100 mM Tris-Cl (pH 8.5), 0.25 mg/ml Naphthol AS-BI phosphate (Sigma) and 0.25 mg/ml fast red violet LB salt (Sigma).

### Embryoid body formation and in vitro differentiation

Clumps of iPSCs were transferred to non-adherent polystyrene dishes containing the ES medium without basic FGF to form embryoid bodies (EBs). The medium was replaced every other day. After 8 days of floating culture, EBs were transferred onto gelatin-coated plates and were maintained in DMEM supplemented with 10% FBS, β-mercaptoethanol, and antibiotics for another 8 days. For detection of senescence phenotypes during differentiation, Y-27632-treated iPSCs were dissociated into single cell suspensions with Accutase (Innovative Cell Technologies) and 1×10^4^ cells were transferred into 96-well V-shaped bottom plates (Greiner Bio-One) to form evenly sized EBs. After 12 days of EB formation in the ES medium without basic FGF, EBs were cultured in DMEM supplemented with 10% FBS, β-mercaptoethanol, and antibiotics.

### Teratoma formation

After harvest, 1×10^6^ iPSCs were injected into the testes of a severe combined immunodeficient (SCID) mice (CREA, Japan). Three months after injection, tumors were dissected and were fixed using 4% paraformaldehyde. Subsequently, dissected tumor tissues were embedded in paraffin and were sliced and stained with hematoxylin and eosin.

### Western blot

Whole cell lysates were prepared in SDS sample buffer and subjected to electrophoresis on 8% SDS-polyacrylamide gels, and separated proteins were transferred onto PVDF membranes (FluoroTrans W, Pall Corporation). Membranes were blocked with TBS-T containing 5% skim milk and were then incubated with anti-WRN (1∶500, 4H12, Abcom) or anti-β-actin (1∶30000, Ac-15, Sigma) monoclonal antibodies for 3 h at room temperature. Membranes were then washed with TBS-T and were incubated with horseradish peroxidase-conjugated anti-mouse IgG (1∶5000, NA931V, GE) for 1 h at room temperature. Chemiluminescence reactions were performed using Western Lightning Plus-ECL (PerkinElmer) and were detected using exposure of x-ray films.

### Mutation analysis

The DNA fragments mut.4 (c.3139-1G>C) and mut.6 (c.1105C>T) were amplified with the primer pairs WS_mut4_U, GGTAAACGGTGTAGGAGTCTGC and WS_mut4_L, CTTGTGAGAGGCCTATAAACTGG, and WS_mut6_U, TGAAGATTCAACTACTGGGGGAGTAC and WS_ mut6_L, ACGGGAATAAAGTCTGCCAGAACC, respectively, using genomic DNA as a template. Mutations were analyzed by direct sequencing using these PCR primers.

### Short tandem repeat (STR) analysis

Genomic DNAs were purified from WS fibroblasts and their derivative iPSC clones using phenol/chloroform extraction and were then used for analysis using a Cell ID System (Promega). PCR products were analyzed using an Applied Biosystems 3130xl Genetic Analyzer and GeneMapper software.

### Gene expression profiling

Cy3-labeled total RNAs were hybridized onto Human Genome U133 Plus 2.0 Arrays (GeneChip, Affymetrix). Arrays were then scanned using the GeneChip Scanner 3000 7G (Affymetrix), and the obtained data were analyzed by Affymetrix Expression Console Software. The microarray dataset has been deposited in the NCBI Gene Expression Omnibus database under Series Accession GSE62114.

### Measurement of telomere length

Genomic DNAs were digested using *Hinf*I restriction enzyme (TakaraBio), and were subjected to electrophoresis on 1% agarose gels. Size-fractionated DNAs were transferred onto Hybond-N+ membranes (GE). Membranes were hybridized with a digoxigenin-labeled (CCCTAA)_4_ probe, and TRFs were detected using TeloTAGGG Telomere Length Assays (Roche Applied Science) according to the manufacturer's instructions.

### RT-PCR and real-time qRT-PCR analysis of mRNA expression

Total RNA was prepared using RNeasy spin columns (Qiagen) according to the manufacturer's instructions. RT-PCR was performed with 0.1 µg of total RNA using SuperScript One-Step RT-PCR (Invitrogen). Semi-quantitative analysis was performed after converting total RNA into cDNA using a High Capacity RNA-to-cDNA kit (Life Technologies), and real-time PCR was performed using a Rotor-Gene SYBR Green PCR kit (Qiagen). Relative gene expression levels were analyzed according to the ΔΔCt method using Ct values of GAPDH mRNA as an internal control. Primer sequences are listed in Tables S1 and S2.

### Immunofluorescence cytochemistry

Following fixation of iPSCs and differentiated cells with 4% paraformaldehyde for 15 min at 4°C, cells were permeabilized with 0.1% Triton X-100, washed with PBS containing 2% BSA, and incubated with primary antibodies diluted in PBS containing 2% BSA.

Primary antibodies against Nanog (1∶200, Cell Signaling, D73G4), SSEA-4 (1∶200, Cell Signaling, MC813), Tra-1-60 (1∶200, Cell Signaling, #4746), Tra-1-81 (1∶200, Cell Signaling, #4745), βIII-tubulin (1∶200, Millipore, TU-20), desmin (1∶200, Neomarkers, RB-9014-P0), vimentin (1∶200, Santa Cruz, V9), and α-fetoprotein (1∶500, Sigma, HPA010607) were detected using the secondary antibodies Alexa 488-conjugated anti-goat IgG (1∶500, Invitrogen, A11055), Alexa 488-conjugated anti-mouse IgG (1∶500, Invitrogen, A11001), Alexa 488-conjugated anti-mouse IgM (1∶500, Invitrogen, A21042), and Alexa 488-conjugated anti-rabbit IgG (1∶500, Invitrogen, A11013). Cell nuclei were stained with 1- µg/ml 4',6-diamidino-2-phenylindole (DAPI).

### Karyotype analysis

After culturing iPSCs in the ES medium containing 100-ng/ml colcemid for 5 h at 37°C, cells were harvested using trypsin and were treated with 0.075 M KCl for 15 min at 37°C. Cells were then fixed in Carnoy's fluid, and chromosome slides were prepared. G-banding analysis was conducted using a previously described method [24].

M-FISH was performed with the Multi-color probe kit “24XCyte” (MetaSystems, Altlussheim, Germany) according to the manufacturer's protocol with slight modifications. Briefly, probes were denatured at 75°C for 5 min and were hybridized to metaphase spreads, which were denatured in 0.07 N NaOH at room temperature for 1 min. Slides were then incubated at 37°C for 2 nights and were then washed in 0.4× SSC at 72°C for 2 min, in 2× SSC containing 0.05% Tween 20 at room temperature for 30 s, and in 2× SSC at room temperature for 1 min, and the mounting medium (DAPI, 125 ng/ml) and a cover slip were applied. Acquisition and analysis of M-FISH images were performed using a CytoVision ChromoFluor System (Applied Imaging, Newcastle upon Tyne, UK).

### Transduction of hTERT gene

PT67 retrovirus packaging cells (Takara Bio USA, Madison, WI, USA) were transfected with pMSCV-hTERT-puro using GenePorter II according to the manufacturer's protocol. After 24 h, the culture medium was replaced, cells were incubated for a further 24 h period, and viral supernatants were harvested, A0031 and WSCU01 WS fibroblasts were infected with viral supernatant in the presence of 8 µg/ml polybrene. Confluent infected cells were then split into 2 new dishes, and puromycin selection of infected cells was initiated at the following passage. Confluent infected cells were then passaged in 4-fold dilutions, leading to an increase in 2 population doubling levels for each passage.

### SA-β-gal assay

SA-β-gal staining was performed as described by Debacq-Chainiaux et al. [25].

### Ethical statement

This study was approved by the Ethics Review Board of the Graduate School of Medicine, Chiba University and was conducted in accordance with the Declaration of Helsinki. Written informed consents were obtained from patients prior to tissue harvesting and iPSC generation, and patients were entitled to the protection of confidential information. Genome/gene analyses performed in this study were approved by the Ethics Committee for Human Genome/Gene Analysis Research at Hiroshima University. All animal experiments were performed in strict compliance with the protocol approved by the Institutional Animal Care and Use Committee of Tottori University (13-Y-18), and the Animal Care and Use Committee of Chiba University (25–131). All recombinant DNA experiments were performed in strict conformance with the guidelines of the Institutional Recombinant DNA Experiment Safety Committee at Hiroshima University.

## Results

### Infinite proliferative potential of WS iPSCs after long-term culture

To determine whether reprogramming provides WS cells with infinite proliferative potential, we generated iPSCs from WS patient fibroblasts. Morphologically distinct colonies from parental cells emerged after transduction of Yamanaka factors using retroviruses and showed elevated alkaline phosphatase activity (Figures S1A and S1B). Colonies were picked up, and 6 WS iPSC lines were established using fibroblasts from 2 independent WS patients after several passages. In western blotting analysis using an anti-WRN antibody, WRN protein was not detected in WS iPSCs but was expressed in both normal fibroblasts and iPSCs (Figure S2A). Direct sequencing analysis of WS iPSCs identified compound heterozygous Mut4/Mut6 mutations in the *WRN* gene similar to those observed in parental cells, and the derivation of WS iPSCs from parental cells was confirmed by STR analysis (Figures S2B and S2C). Finally, the 6 WS iPSC lines #23, #34, and #64 from A0031 and #02, #13, and#14 from WSCU01 were successfully established.

**Figure 1:**
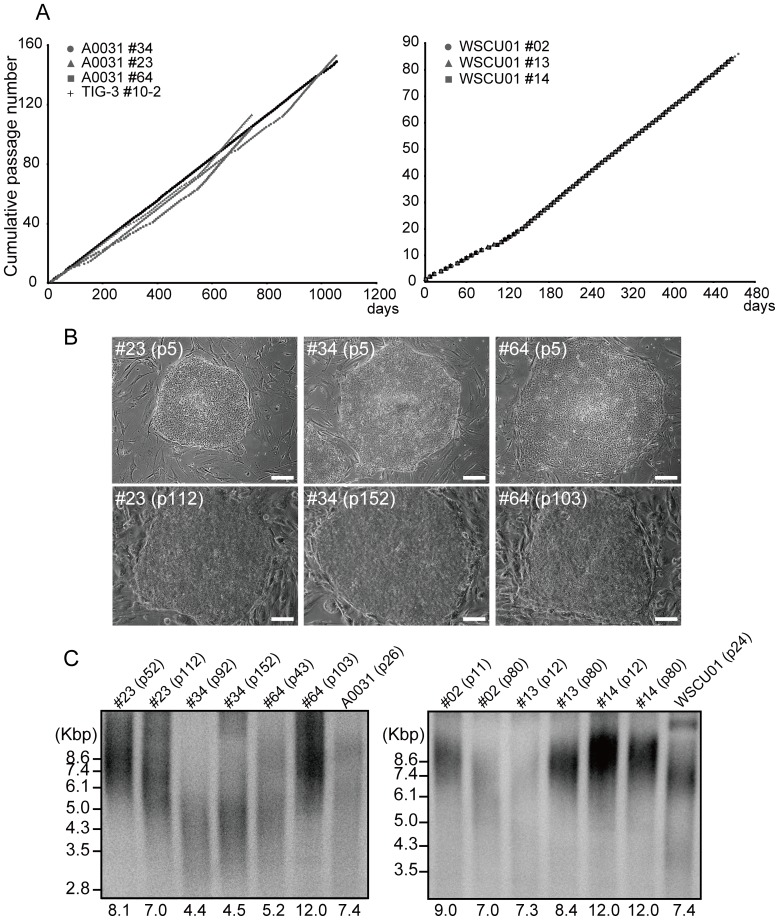
Infinite Proliferation of WS iPSCs after Long-Term Culture. (A) Cumulative passage number for WS iPSCs. (B) Colony morphologies of A0031-derived WS iPSC clones in early and late passages. Bars  = 100 µm. (C) TRF length analysis of WS iPSC clones in early and late passages.

WS iPSC lines from A0031 were cultured for 120 continuous passages over 2 years without morphological changes or loss of growth capacity (Figures 1A and 1B). Moreover, iPSC lines from WSCU01 proliferated for a year (Figures 1A and S1C). Average terminal restriction fragment (TRF) lengths in clones #23, #34, and #64 (A0031) were decreased, invariable, and increased during long-term culture, respectively, and similar telomere dynamics were observed in WSCU01-derived iPSC clones (Figure 1C).

### Sustained ESC-like characters of WS iPSCs after long-term culture

**Figure 2:**
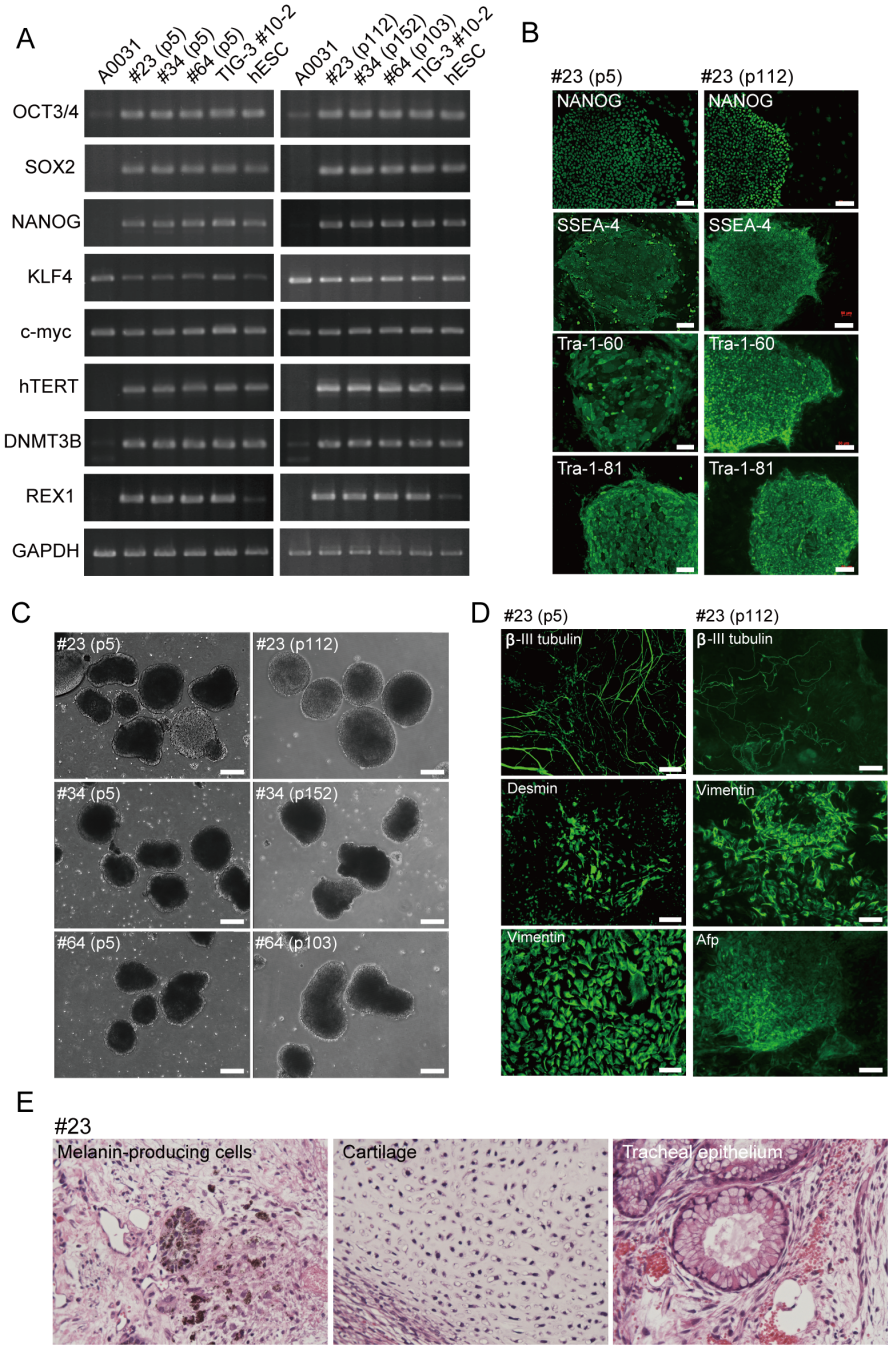
Sustained ESC-like characteristics of WS iPSCs after Long-Term Culture. (A) Expression of pluripotency genes in A0031-derived WS iPSC clones in early and late passages. (B) Expression of hESC markers in A0031-derived WS iPSC clone #23 in early and late passages. Bars  = 100 µm. (C) EB formation in A0031-derived WS iPSC clones from early and late passages. Bars  = 100 µm. (D) Immunocytochemical analysis of differentiation of EBs into 3 germ layers for A0031-derived iPSC clone #23 in early and late passages. β-III tubulin (ectoderm), desmin (mesoderm), vimentin (mesoderm and parietal endoderm), and α-fetoprotein (Afp, endoderm). Bars  = 100 µm. (E) Hematoxylin and eosin histology of teratomas from A0031-derived iPSC clone #23. Formation of all 3 germ layers is shown including melanin-producing cells (ectoderm), cartilage (mesoderm), and tracheal epithelium (endoderm).

To determine the persistence of ESC-like characteristics in WS iPSCs, we compared undifferentiated states and differentiation potentials between WS iPSCs from early and late passages. WS iPSC lines expressed pluripotency genes and hESC-specific surface markers during early passages (around p10), and during late passages (around p100; Figures 2A, 2B, S3 and S4). These iPSC lines also showed sustained formation of embryoid bodies and differentiation into 3 germ layers (Figures 2C, 2D, and S5). Furthermore, at around p50, WS iPSC lines generated teratomas that contained tissue structures of all 3 germ layers. These were consistent with those shown in normal iPSC lines after transplantation into the testes of SCID mice (Figures 2E and S6). Thus, reprogrammed WS fibroblasts acquired infinite proliferative potential, and the ESC-like characteristics of the resulting iPSCs were maintained for more than 2 years.

### Suppression of senescence-associated gene expression in WS iPSCs after long-term culture

**Figure 3:**
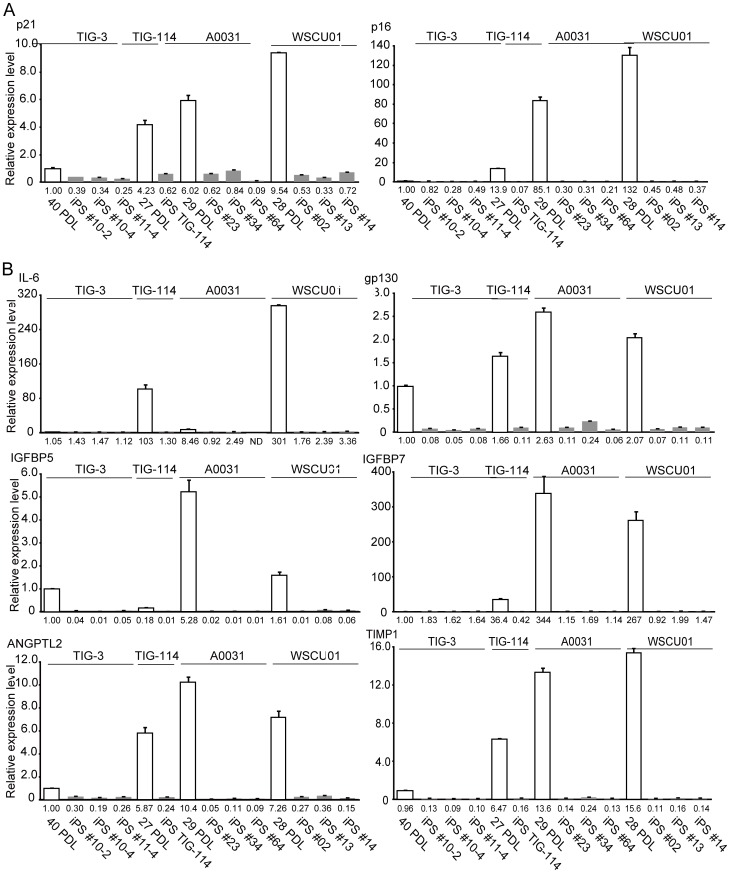
Suppression of Senescence-Associated Gene Expression in Reprogrammed WS iPSCs. (A) Expression of CDKI genes in parental fibroblasts and iPSCs. White columns show relative expression levels in the parental fibroblasts TIG-3, TIG-114, A0031, and WSCU01, and gray columns show those of their derived iPSC clones. Numbers under the horizontal axis in each graph show relative values in mRNA expression compared with that in TIG-3 fibroblasts. Values represent means of three technical replicates ± SD. (B) Expression of SASP genes in parental fibroblasts and iPSCs. Each graph is shown as in (A).

Global gene expression analysis using DNA chips showed pronounced similarities among pluripotent stem cells including WS iPSCs. However, marked differences between WS iPSC and WS fibroblasts were observed (Figure S7). Heat map analysis also showed a high analogy of global gene expression profiles in these pluripotent stem cell lines, but distinctly different profiles from those of WS fibroblasts (Figures S8A). Recent studies of aging have identified senescence-induced inflammatory and secretory factors that are collectively referred to as the senescence-associated secretory phenotype (SASP) and are the hallmarks of aging. It is widely accepted that age-associated inflammatory responses contribute to human aging mechanisms [26]. Accordingly, we observed downregulation of SASP secretory factors, including inflammatory cytokines, growth factors and MMPs, in both normal and WS iPSCs compared with WS fibroblasts (Figures S8B). Subsequently, we performed real-time qRT-PCR analysis using PDL-matched normal and patient fibroblasts, and their iPSC derivatives which were maintained in long-term culture. Although relative expression levels of the senescence-associated cyclin-dependent kinase inhibitor (CDKI) genes *p21Waf1/Cip1* and *p16INK4a* in normal fibroblasts correlated with the donor age, the expression levels of these genes were higher in WS fibroblasts than in normal fibroblasts, indicating that replicative senescence was prematurely induced in WS cells (Figure 3A). However, expression levels of these genes were significantly reduced in all iPSC clones from normal and WS cells (Figure 3A), suggesting that these gene loci are reprogrammed to the same degree in normal and WS iPSCs. Thus, we examined the expression of the typical SASP genes *IL-6* and *gp130* [27] and found higher expression levels in WS fibroblasts than in normal fibroblasts (Figure 3B). Moreover, expression levels of these genes drastically decreased in both normal and WS iPSCs compared with parental fibroblasts. Similarly, expression levels of the SASP genes *IGFBP5*, *IGFBP7*, *ANGPTL2*, and *TIMP1* ([28]–[31] were significantly decreased in both normal and WS iPSCs compared with parental fibroblasts (Figures 3B).

### Reprogramming of the SASP gene loci is mediated by factors other than activated telomerase

**Figure 4:**
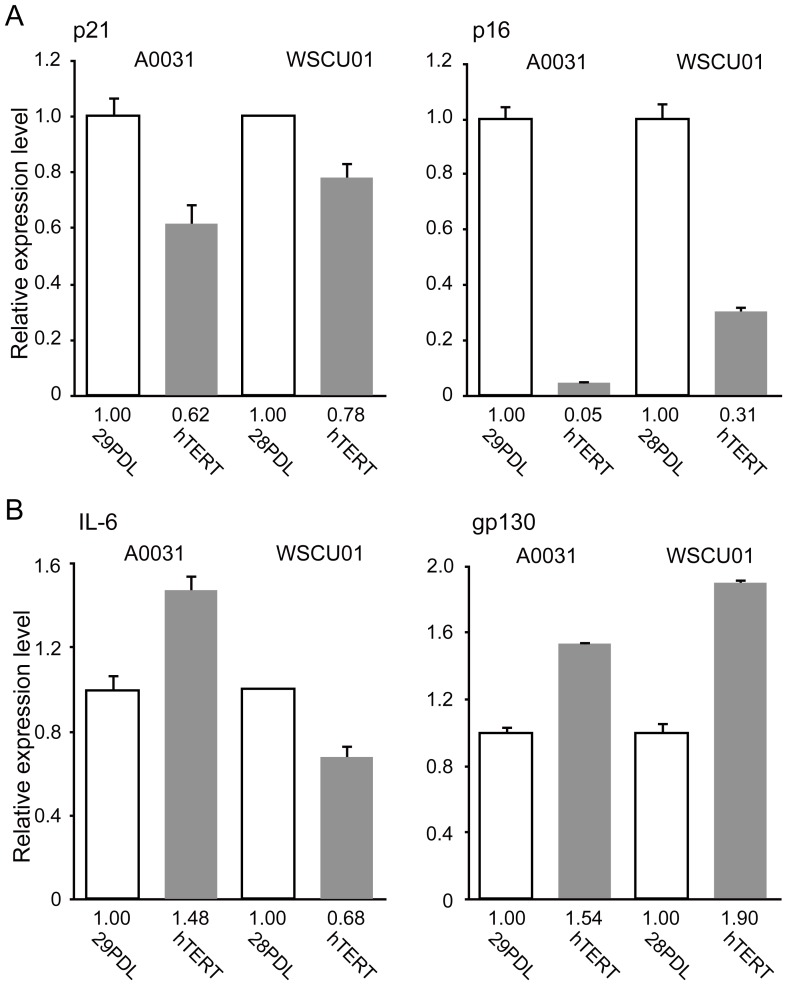
Reprogramming of the SASP gene loci is mediated by factors other than activated telomerase. (A) Expression of CDKI genes in WS fibroblasts and their hTERT-transduced derivatives. White columns show relative expression levels in A0031 and WSCU01 fibroblasts, and gray columns show those of their hTERT-transduced derivatives. Numbers under the horizontal axis in each graph show relative values in mRNA expression compared with that in parental fibroblasts. Values represent means of three technical replicates ± SD. (B) Expression levels of SASP genes in WS fibroblasts and their hTERT-transduced derivatives. Each graph is shown as in (C).

WS fibroblasts were previously shown to bypass premature senescence following introduction of the telomerase gene *hTERT* [32], Similarly, the present WS cells bypassed premature replicative senescence, and hTERT allowed cell division for over 150 PDL in A0031 cells, and 40 PDL in WSCU01 cells compared with parental cells that became senescent at less than 30 PDL (Figures S9A and S9B). TRF length analysis showed that hTERT-expressing WS cells acquired longer telomeres during passages than parental cells (Figures S9C). To examine whether the expression of hTERT was sufficient to suppress the upregulation of aging-associated genes in WS cells, we compared expression levels of CDKI and SASP genes between WS fibroblasts and their hTERT-expressing derivatives. Whereas a decline in p21waf1/cip1 and p16INK4a mRNA expression was observed in hTERT-expressing cells (Figure 4A), IL-6 and gp130 expression was not suppressed following the introduction of hTERT, suggesting that reprogramming of the SASP gene loci is mediated by factors other than activated telomerase (Figure 4B). The present data show complete suppression of premature senescence phenotypes in WS cells using transcription factor-induced reprogramming and suggest that persistence of the undifferentiated state and pluripotency are crucial for reversing the aging process.

### Recapitulation of premature senescence phenotypes in differentiated cells from WS iPSCs

**Figure 5:**
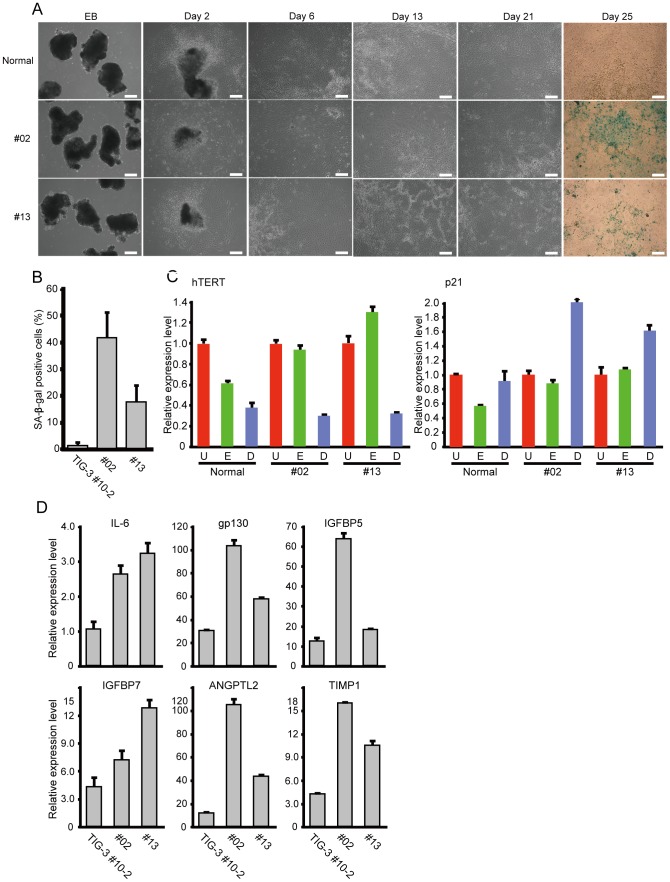
Recapitulation of Premature Senescence Phenotypes in Differentiated Cells from WS iPSCs. (A) Differentiation of EBs from normal (TIG-3) and WS (WSCU01 #02 and #13) iPSCs. Differentiated cells from WS iPSCs showed premature senescence. SA-β-gal staining was performed on day 25 of differentiation. Bars  = 100 µm. (B) Percentage of senescent cells after 25 days of differentiation. SA-β-gal-positive cells were counted in three randomly selected fields with 40× magnification. Values represent means of the three fields ± SD. (C) Expression of hTERT and p21 mRNAs in undifferentiated iPSCs (“U,” red columns), EBs after 12 days of formation (“E,” green columns), and differentiated cells after 25 days of differentiation (“D,” blue columns). Values represent means of three technical replicates ± SD. (D) Expression of SASP genes in differentiated cells from normal (TIG-3) and WS (WSCU01 #02 and #13) iPSCs after 25 days of differentiation. Graphs shows fold changes relative to undifferentiated iPSCs. Values represent means of three technical replicates ± SD.

To establish cell lineages that prematurely senesced, EBs consisting of equal numbers of iPSCs maintained in long-term culture were differentiated in serum-containing medium. Differentiated cells from WS iPSC-derived EBs were outgrown less rapidly than those from normal iPSC-derived EBs (Figure 5A, Day 2). These cells exhibited flat and enlarged morphology (Figure 5A, Day 6, 13, and 21) and became positive for SA-β-gal staining (Figure 5A, Day 25, and Figure 5B). Whereas expression levels of hTERT were downregulated equally in differentiated cells from normal and WS iPSCs, p21 mRNA was more highly induced in differentiated cells from WS iPSCs than those from normal iPSCs (Figure 5C). Expression levels of the SASP genes were also significantly increased in differentiated cells from WS iPSCs compared with those from normal iPSCs (Figure 5D). These results demonstrated recapitulation of premature senescence phenotypes with downregulation of hTERT in differentiated cells from WS iPSCs.

### Karyotype analysis of WS iPSCs

WS is characterized by genomic instability, and gene translocation events have been observed during culture of patient-derived cells [33]. Because reprogramming of somatic cells and subsequent maintenance of iPSCs involves extensive cell division, WS iPSCs may acquire additional chromosomal abnormalities. Thus, we compared chromosomal profiles of long-term cultured WS iPSC clones with those of parental WS fibroblasts by karyotype analysis. The subsequent G-banding stain and multicolor fluorescence *in situ* hybridization (M-FISH) analysis are summarized in Table 1.

**Table 1:** Results of chromosome analysis of WS iPSC clones and their parenral cells.

Cell lines	Numbers of cells analyzed by G-banding	Numbers of cells analyzed by M-FISH	Karyotypes
A0031	20 (13/7)	ND	46,XY,del(8)(q22q24)/46,XY,t(1;14)(p34.1;q13),t(4;7)(p15.2;q22),del(8)(q22q24)
iPS#23	20	10	46,XY,t(1;14)(p34.1;q13),t(4;7)(p15.2;q22),del(8)(q22q24),der(21)t(17;21)(?;q22.3)
iPS#34	20	10	46,XY,t(1;14)(p34.1;q13),t(4;7)(p15.2;q22),del(8)(q22q24)
iPS#64	20	10	46,XY,t(1;14)(p34.1;q13),t(4;7)(p15.2;q22),del(8)(q22q24),der(19)t(2;19)(?;p13.3)
WSCU01	20	ND	46,XY,normal
iPS#02	20	10	47,XY,+del(20)(p?)
iPS#13	20	10	46,XY,normal
iPS#14	20	10	46,XY,normal

Abbreviations: t, translocation; del, deletion; der, derivative chromosome; p, short arm; q, long arm.

**Figure 6:**
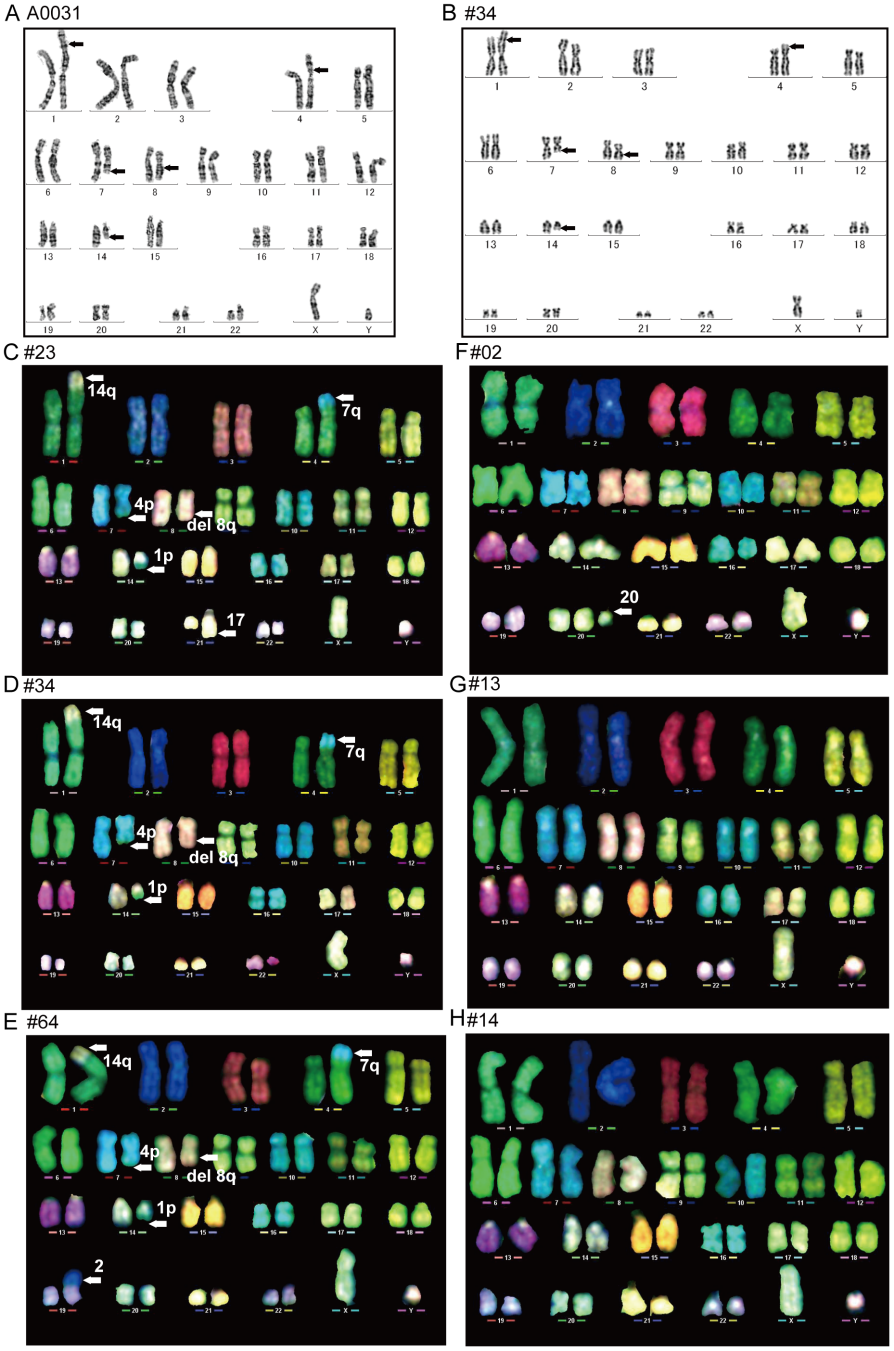
Karyotype Analysis of WS iPSCs. Chromosomal profiles of G-band analysis. (A) Parental A0031 fibroblast and (B) A0031-derived iPSC clone #34. Arrows indicate translocation breakpoints. Chromosomal profiles of M-FISH analysis. A0031-derived iPSC clones (C) #23, (D) #34, and (E) #64 and WSCU01-derived iPSC clones (F) #02, (G) #13, and (H) #14. Arrows indicate translocation breakpoints or an extra chromosome.

Chromosomal profiles of parental A0031 WS fibroblasts showed mosaicism with the following abnormal karyotypes: 46, XY with a deletion in 8q and 46, XY with a deletion in 8q along with reciprocal translocations between 1p and 14q, and 4p and 7q (Figure 6A). These karyotypes support previous observations of chromosomal instability in WS cells [33]. Whereas, 1 of the derived iPSC clones (#34) had the same chromosomal profile as its parent cells (Figures 6B and 6D), the other 2 A0031-derived iPSC clones (#23 and #64) had the translocations 21q and 19p, respectively, in addition to those of the parental karyotype (Table 1, Figures 6C and 6E). Moreover, whereas parental WSCU01 fibroblasts and 2 of their derived iPSC clones (#13 and #14) had normal karyotypes (Table 1, Figures 6G and 6H), the remaining iPSC clone #02 carried the abnormal karyotype 47 (XY with an additional aberrant chromosome derived from chromosome 20; Table 1, Figure 6F).

The observation that 3 of 6 WS iPSC clones had the same karyotypes as their parental cells after approximately 100 passages suggests that karyotypes of WS cells are stabilized following reprogramming.

## Discussion

In this study, we demonstrated that WS fibroblasts could be reprogrammed into iPSCs using Yamanaka factors, and the resulting iPSCs showed unlimited proliferative capacity that was sufficient for self-renewal over a period of 2 years. WS iPSCs also exhibited undifferentiated states and differentiation potential after long-term culture. Subsequently, we showed that WS iPSCs maintain immortality and ESC-like characteristics that indicate corrected telomere dysfunction following reprogramming of WS cells. Although WRN was not essential for generation of iPSCs, WRN helicase may protect genome integrity by mechanism other than the maintenance of telomere in iPSCs.

TRF length analysis indicated that WS iPSC lines maintained telomere with size variation in each clone. It is known that human iPSCs derived from normal somatic cells showed varied telomere length, and variation of telomere length among human iPSC clones is thought to partly depend on acquired telomerase activity associated with their reprogrammed states [34], [35]. Therefore, variation of telomere length observed among WS iPSC clones would be due to clonal variation in telomerase activity rather than telomere dysfunction associated with *WRN* deficiency.

Normal human iPSCs are known to acquire genomic instability with a high incidence of additions, deletions and translocations [36], [37]. In contrast, chromosomal aberrations are frequently caused by telomere dysfunctions in WS fibroblasts following the induction of cell cycle progression [11]. Nonetheless, the present data show unexpected maintenance of chromosomal profiles in WS iPSC clones during long-term culture for more than 100 passages although half of these clones acquired additional chromosomal abnormalities. Previously, the introduction of hTERT reduced the chromosomal aberrations in cells from WS patients [11]. In agreement, the present data indicate endogenous hTERT expression in WS iPSCs, but not in parental fibroblasts, suggesting that reprogramming suppresses chromosomal instability in WS cells by reactivating telomerase.

Previous studies show that WS fibroblasts express inflammatory cytokines [38] and WS is associated with inflammatory conditions such as atherosclerosis, diabetes and osteoporosis [39]–[43]. The present data indicate that both CDKI and SASP genes are prematurely induced in WS fibroblasts compared with PDL-matched normal fibroblasts. However, expression levels of these genes were completely suppressed in WS iPSCs to the same degree observed in normal iPSCs. In contrast, hTERT did not suppress SASP genes in WS fibroblasts, as shown by previous study [44], although a decline in p21waf1/cip1 and p16INK4a mRNAs was observed. Taken together, these observations suggest that pluripotency-associated transcription factor-induced reprogramming reverses the aging process in both normal and WS cells. Furthermore, differentiated cells from EBs of long-term cultured WS iPSCs showed premature senescence phenotypes, thus demonstrating that WS iPSCs stably maintained their potential to recapitulate premature senescence phenotypes during differentiation over the long term. In addition, embryoid body-mediated iPSC differentiation recapitulated premature senescence phenotypes in WS iPSCs, suggesting that it would provide a simple and rapid way to identify cell lineages affected in WS.

In the present study, we demonstrated the potential of WS iPSCs to proliferate infinitely and differentiate into various cell types, which could be used to provide patient cells in large quantities over the long term. Because WS-specific iPSCs may be differentiated into multiple cell types, their experimental use may resolve the major pathogenic processes of WS for which cell types available from patients are usually limited to lymphocytes and/or fibroblasts. The present technologies may also be used to develop cell transplantation therapies for WS patients using gene-corrected patient cells. The present observations indicate that WS iPSCs may be a powerful tool for understanding normal aging and the pathogenesis of WS.

## Supporting Information

Figure S1 **Generation of WS iPSCs.** (A) Generation of iPSCs. Normal (TIG-3) and Werner syndrome (A0031 and WSCU01) fibroblasts are shown in the left panels, and emergence of morphologically distinct ESC-like colonies from parental cells is shown in the right panels. (B) Alkaline phosphatase activity of ESC-like colonies derived from TIG-3 and A0031 fibroblasts. (C) Colony morphologies of WSCU01-derived WS iPSC clones in early and late passages. Bars  = 100 µm.
(EPS)

Figure S2 **Evidences that WS iPSCs were derived from patients.** (A) Western blot analysis of WRN helicase protein in WS iPSCs. (B) Direct sequencing analysis identified compound heterozygous mut.4/mut.6 mutations in WS iPSCs. Mut.4 is a C to G substitution at the splice-donor site bordered by exon 26, as shown by an arrow in the illustration of the double-strand base sequence. Obtained pherograms show antisense peak shapes. A peak corresponding to mut.4 in normal TIG-3 fibroblast shows a single “C,” whereas the WS iPSC clone #34 from A0031 fibroblasts gave double peaks showing “G” in addition to “C.” Mut.6 is a T to C substitution in exon 9. A peak corresponding to mut.6 in normal cells showed a single “C,” whereas WS iPSC gave double peaks showing “T” in addition to “C.” C, blue; G, black; T, red; A, green. (C) STR analysis of A0031-derived iPSC clone #34, showing that iPSC clone #34 was derived from the parental A0031 fibroblasts.
(EPS)

Figure S3 **Expression of pluripotency genes in WSCU01-derived WS iPSC clones in early and late passages.**
(EPS)

Figure S4 **Expression of hESC markers in WS iPSCs in early and late passages.** A0031-derived clones #34, and #64, and WSCU01-derived clones #02, #13, and #14 are shown. Bars  = 100 µm.
(EPS)

Figure S5 **Immunocytochemistry for differentiation of embryoid bodies into 3 germ layers for WS iPSCs in early and late passages.** A0031-derived clones #34, and #64, and WSCU01-derived clones #02, #13, and #14 are shown. Bars  = 100 µm.
(EPS)

Figure S6 **Hematoxylin and eosin histology of teratomas derived from iPSCs.** Hematoxylin and eosin histology of teratomas derived from iPSCs. The normal TIG-3 fibroblast-derived clone #10-2, A0031-derived clones #34, and #64, and the WSCU01-derived clone #02 are shown. Formation of all 3 germ layers is shown with melanin-producing cells and glial tissue (ectoderm), cartilage (mesoderm) and intestinal epithelia. Glands are lined by columnar epithelia and tracheal epithelium (endoderm).
(EPS)

Figure S7 **Figure Scatter plots comparing gene expression profiles.**
(EPS)

Figure S8 **Analysis of senescence-associated gene expression in iPSCs.** (A) Heat map analysis of WS iPSC #34 and parental WS A0031 fibroblasts, normal TIG-3 fibroblast-derived iPSCs, and hESC; 3277 probes with >5-fold differences in expression between A0031 fibroblast and WS iPSC were included in the heat map. (B) Heat map analysis of the gene profiles of secreted protein probes with >2-fold differences in expression between A0031 fibroblasts and the 3 pluripotent stem cell lines WS iPSC, TIG-3 iPSC, and hESC.
(EPS)

Figure S9 **hTERT bypassed premature replicative senescence of WS fibroblasts.** (A) Morphologies of growing normal TIG-3 fibroblasts, and A0031 and WSCU01 WS fibroblasts. WS fibroblasts showed premature senescence. SA-β-gal staining was performed for WSCU01 (lower). Bars  = 100 µm. (B) Cumulative population doubling levels for hTERT-expressing WS cells. (C) TRF lengths of A0031 fibroblasts and their TERT-transduced derivatives.
(EPS)

Table S1 (EPS)

Table S2 (EPS)
